# Tubular adenoma with high-grade dysplasia in the ileal segment 34 years after augmentation ileocystoplasty: report of a first case

**DOI:** 10.1186/1746-1596-2-29

**Published:** 2007-08-13

**Authors:** Henry B Armah, Alyssa M Krasinskas, Anil V Parwani

**Affiliations:** 1 Department of Pathology, University of Pittsburgh Medical Center, Pittsburgh, PA, USA

## Abstract

Neoplasms of the urinary bladder following augmentation ileocystoplasty are rare. We present the case of a 39-year-old male with a tubular adenoma with high-grade dysplasia in the ileal segment 34 years after augmentation ileocystoplasty to enlarge a post-chemoradiation-induced shrunken bladder. He presented with gross hematuria. Cystoscopy revealed a papillary tumor at the site of ileovesical anastomosis, and transurethral resection was performed. Histologic examination revealed a tubular adenoma with high-grade dysplasia. There are only two previous reports of tubulovillous adenoma in ileal segment after ileocystoplasty, both without high-grade dysplasia. Our observation supports the hypothesis that an ileal neobladder may undergo all the morphologic and molecular changes observed in the development of gastrointestinal adenocarcinoma. Therefore, patients who had an ileal neobladder created should be closely followed.

## Background

Currently, the use of intestinal segments is the main option both for augmentation and replacement of the urinary bladder, and for diversion of urine from the normal outlet in patients following cystectomy or post-therapy shrunken bladder. These surgical procedures are separated into two main groups, namely bladder substitution or augmentation with transposed intestinal segments and ureterosigmoidostomy. Several types of orthotopic bladder substitution or augmentation have been developed, of which ileocystoplasty with the creation of an ileal neobladder is one of the most common procedures. An ileal neobladder is easily constructed and provides unchanged voiding habits with good continence and upper urinary tract preservation, with relatively low rates of complication [1]. Ureterosigmoidostomy is the surgical procedure where the ureters are diverted into the fecal stream of the sigmoid colon to allow the anal sphincter to maintain both bowel and urinary continence.

There is now a general consensus from long-term follow-up studies that ureterosigmoidostomy results in an increased absolute risk of adenocarcinoma arising from the ureterocolic anastomosis [1-5]. There is at present insufficient conclusive evidence that ileocystoplasty is responsible for an increased absolute risk of malignancy [1]. A recent Cochrane meta-analytic review found few prospective, randomized or case-control studies involving the use of intestinal segments and none of these were designed to assess the risk of secondary malignancy [6]. However, to date, there have been 10 reported cases of carcinomas developing in the ileal segment after ileocystoplasty in the English language medical literature [7-9]. There has been only 1 previous report of a tubulovillous adenoma arising in the cecal segment after cecocystoplasty, without high-grade dysplasia [10]. Finally, there have been only 2 previous reports of tubulovillous adenoma developing in the ileal segment after augmentation ileocystoplasty, both without high-grade dysplasia [11,12]. We report the first case of a tubular adenoma with high-grade dysplasia that developed in the ileal segment 34 years after augmentation ileocystoplasty.

## Case presentation

**Figure 1:**
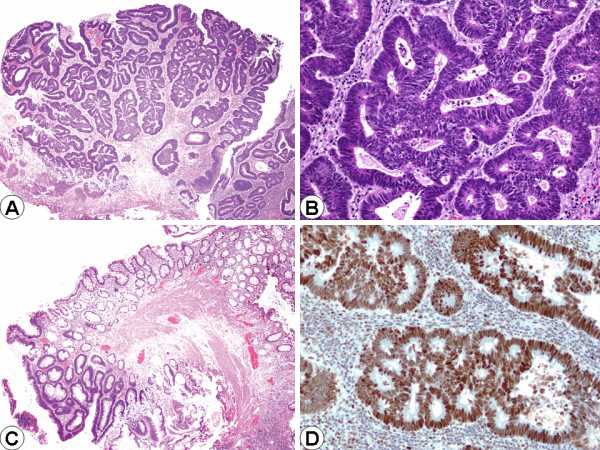
(A) Tubular adenoma with high-grade dysplasia (H&E, Original magnification × 40). (B) Tubular adenoma with high-grade dysplasia (H&E, Original magnification ×100). (C) Tubular adenoma with adjacent colonic metaplasia (H&E, Original magnification × 40). (D) Both MSH2 (shown) and MLH1 (not shown) demonstrated intact (positive) nuclear staining in the dysplastic ileal epithelium (Immunoperoxidase, Original magnification × 200).

A 39-year-old man underwent augmentation ileocystoplasty 34 years previously to enlarge his shrunken bladder following chemotherapy and radiation therapy to his bladder and prostate for embryonal rhabdomyosarcoma at the age of 5. He was completely continent and was voiding normally after the augmentation ileocystoplasty. He had no family history of gastrointestinal malignancy. He presented with gross hematuria. A voiding cystogram showed a polypoid mass at the level of the ileovesical anastomosis. An intravenous pyelogram showed no evidence of a mass or obstruction in the upper urinary tract. Computerized tomography scans showed normal kidneys without obstruction. Flexible cystoscopy revealed a solitary 5 cm papillary tumor of the ileal segment in close proximity of the site of ileovesical anastomosis. Transurethral loop resection was used to completely excise the tumor to the underlying muscle. Histopathologic examination revealed a tubular adenoma with high-grade dysplasia (Figures 1A &1B), and the adjacent ileal mucosa showed features of colonic metaplasia (Figure 1C). Based on the patients's age of 39 (less than 50 years old), immunohistochemical staining for the major DNA mismatch-repair (MMR) proteins MLH1 and MSH2 was performed to exclude the possiblity ofan inherited form of intestinal neoplasia, hereditary non-polyposis colorectal cancer (HNPCC). Immunohistochemical staining for MSH2 and MLH1 revealed that the high-grade dysplastic ileal epithelial cells retained both MSH2 (Figure 1D) and MLH1 (not shown) expression, supporting anabsence of microsatellite instability (MSI) in this neoplasm. Clinically, the complete transurethral resection of the tumor was considered adequate conservative management, and he has undergone follow-up cystoscopy at 3-month intervals for the past year with no evidence of recurrence.

## Discussion

Neoplastic changes after ureterosigmoidostomy as urinary diversion are not uncommon, and these neoplasms are generally attributed to the mixture of the urine and fecal stream [1]. Several case series have been published on the long-term follow-up of children with congenital malformation of the lower urinary tract who underwent ureterosigmoidostomy as urinary diversion. There is now a general consensus from these studies that ureterosigmoidostomy results in an increased absolute risk of adenocarcinoma arising from the ureterocolic anastomosis [1-5]. An estimated lifetime incidence of 5% to 19% for malignant neoplasms following ureterosigmoidostomy has been reported in these case series [1-3]. Approximately half of these malignant neoplasms are adenocarcinomas, and they tend to develop at the site of anastomosis. A long latency period for the development of neoplastic change was a consistent finding, with a median of 26 years (range 5 to 50 years) [1,3].

Neoplasms developing after ileocystoplasty are indeed rare; only 10 cases of carcinomas developing after ileocystoplasty have been reported in the English language medical literature [7-9]. However, their characteristics are similar to those developing after ureterosigmoidostomy. Of the 10 cases of carcinomas developing after ileocystoplasty, 8 were adenocarcinoma. In 8 of these 10 cases, the tumor developed at the ileovesical junction, and in the native bladder of the remaining 2 cases. The latent period from the original surgery to the development of cancer ranges from 4 to 32 years [7-9]. The peri-anastomotic location of the tumor in the case herein presented is in keeping with the above reported location of most tumors arising after ileocystoplasty. Though the evidence for an increased absolute risk of malignancy after ileocystoplasty is inconclusive from meta-analytic review [6], our finding of tubular adenoma with high-grade dysplasia and previous reports of adenocarcinomas in the ileal neobladder [7-9] suggests that the development of gastrointestinal malignancy after ileocystoplasty is a constant, low, but still distinct, risk, even in the absence of mixture of the urine and fecal stream. Since an estimated 153,270 new cases of large bowel cancers occurred compared to an estimated 6,170 new cases of small bowel cancers in the year 2006 (an approximately 25-fold difference in incidence) [13], small intestinal urinary derivations are considered to be at low risk of malignancy. Therefore, small bowel segments are being used increasingly in lower urinary tract reconstruction.

Two main hypotheses have been proposed for the increased risk of neoplastic changes following ureterosigmoidostomy and ileocystoplasty [1,14,15]. The first hypothesis, carcinogenic N-nitrosamine hypothesis, states that the excessive production of N-nitrosamines of known high carcinogenicity by the action of bacteria on urinary nitrate activated malignant change [1,14]. N-nitrosamines are produced when urinary nitrates are reduced to nitrites by bacteria, especially by *Escherichia coli*. Nitrites and urinary amines react to form N-nitrosamines. Experimental studies using animal models have reported both supportive [14,16] and contradictory [17,18] evidence for this hypothesis. It is generally believed that malignant change occurring in association with urinary reconstruction using intestinal segments is a result of urinary stagnation and bacteriuria, chronic inflammation and carcinogenic substances, such as N-nitrosamine.

Ureterosigmoidostomy patients have consistently showed substantially higher levels of N-nitrosamine in their faeces or urine compared to control subjects, and was associated with chronic inflammatory changes and altered mucin secretion at the anastomotic site [5]. However, the administration of vitamin C (an inhibitor of N-nitrosamine formation) in a subsequent small preliminary clinical study resulted in only a modest, statistically insignificant, decline in the N-nitrosamine content of rectal slurry [19]. Ileocystoplasty patients showed villous atrophy with chronic inflammation and had high concentration of N-nitrosamine in urine samples compared to control subjects [20]. The levels of N-nitrosamines in the urine of patients after augmentation ileocystoplasty have recently been reported to be greatest among those with continued urinary infection, those with sterile pyuria, those using intermittent catheterization, and those not taking prophylactic antibiotics [21]. The levels of N-nitrosamines within urine from ileal neobladder are intermediate between those found in normal controls and patients with ureterosigmoidostomy [21,22]. Additionally, a significantly higher prevalence of bacteriuria (47%) has been reported in patients after augmentation ileocystoplasty compared to controls [20].

The second hypothesis, inflammatory response hypothesis, states that the inflammatory response stimulated by the anastomosis of differing mucosa lead to nuclear instability, perhaps through the generation of reactive oxygen species [1,14]. However, direct supportive experimental and clinical evidence for this hypothesis are lacking [1,14,15]. One of the most intriguing facts of the previously reported cases of adenocarcinoma or tubular adenomas [7-9] and of the present case of tubular adenoma with high-grade dysplasia following ileocystoplasty is that almost all tumors developed at the anastomotic site and not elsewhere in the intestinal segment. Interestingly, the same situation occurs in patients following ureterosigmoidostomy where most adenocarcinomas developed at the site of the anastomosis [1,3]. Additionally, though spontaneous ileal malignancies are very rare, ileal malignancies have been more frequently reported at the site of surgical anastomosis such as ileo-ileostomy [23]. The inflammatory response hypothesis has been suggested as accounting for the increased susceptibility of development of tumors at the line of anastomosis between the intestinal segment and native bladder, but the supporting evidence is inconclusive and the exact mechanism is not well understood [1,14,15]. We hypothesize that the highest levels of carcinogenic N-nitrosamines are present closest to the anastomosis (on account of stasis or reduced motility at that site postoperatively) coupled with the inflammatory response stimulated by the anastomosis of differing mucosa lead to nuclear instability and the initiation of the metaplasia-adenoma-carcinoma sequence. However, additional long-term clinicohistological and animal studies are required to confirm our hypotheis.

Clinical studies examining the cellular origin of tumors found at the line of anastomosis between the intestinal segment and native bladder have reported contradictory findings [24,25]. Though, one study suggested the origin of the adenocarcinoma from metaplastic bladder mucosa [24], another study suggested that the neoplastic changes were consistent with an intestinal origin on the basis of histochemical staining [25]. In a follow-up prospective study of 24 patients with orthotopic ileal neobladder who underwent regular endoscopic biopsy for 5 years after surgery, progressive changes to the ileal mucosa were seen, with thinning, increased goblet cells, and villous atrophy, but no dysplasia and no malignancy [26]. Three similar studies of patients with orthotopic ileal neobladder (4-year follow-up study of 30 patients [27], 7-year follow-up study of 90 patients [28,29], and 10-year follow-up study of 20 patients [30]) found similar progressive changes to the ileal mucosa, but with no atrophy, no dysplasia and no malignancy. Additionally, histochemical staining revealed the increased presence of mucin (sulfomucins and sialomucins) immediately postoperatively which gradually reduced with extended follow-up [26-30]. These 5 recent follow-up studies suggested that the ileal mucosal changes were adaptive rather than premalignant [26-30].

Therefore, evidence in support of either of the two hypotheses of pathogenesis for malignancies following ureterosigmoidostomy and ileocystoplasty is incomplete and conflicting [1,14-22,26-30]. Recent animal studies do not suggest a significant role for nitrosamines as the carcinogenic agent [14,16-18], whilst histological studies have not shown clear evidence of premalignant mucosal change subsequent to inflammation of the anastomotic site [26-30]. Although nitrosamine excretion and bacterial load is increased in ileocystoplasty patients [20-22], this does not seem to lead to features of premalignant mucosal change during medium-term follow-up. Transposed intestinal segments exposed to urine undergo a well-characterized series of adaptive changes toward a more transitional phenotype over a period of 4–10 years [26-30]. Early inflammatory changes associated with surgery and seen in more acute animal models soon disappear without any evidence of the continued secretion of abnormal sialomucins [26-30]. These progressive changes result in a relatively thin intestinal epithelium with villous atrophy but without dysplastic or neoplastic features, although the extent of sampling of clinical material is necessarily limited for ethical reasons [1,6,28]. However, our finding of tubular adenoma with high-grade dysplasia adjacent to adaptive colonic metaplasia 34 years after ileocystoplasty and the previous report of adenocarcinoma 20 years after ileocystoplasty [7] suggest that the adaptive changes may progress to premalignant and malignant changes. Therefore, the adaptive colonic changes observed up to 10 years in the 5 studies above [26-30] may progress to premalignant and malignant changes in the ileal mucosa as late complications after ileocystoplasty. As with all malignancies, the pathway for carcinogenesis following ureterosigmoidostomy and ileocystoplasty is likely to be complex and multifactorial, and requires additional long-term histological studies to suggest further experimental approaches.

Immunohistochemical, the high-grade dysplastic ileal epithelial cells in the case herein presented expressed both DNA MMR proteins MSH2 and MLH1. The presence of protein expression of these 2 major human DNA MMR proteins indicates the absence of pathogenic mutations in these MMR genes and the absence of microsatellite instability (MSI) at this stage in this neoplastic process. Therefore, MSI was not contributory to the pathogenesis of the high-grade dysplastic adenomatous changes in the case herein presented, but does not exclude the possibility that MSI may contribute to the subsequent progression to invasive adenocarcinoma. Indeed, a recent report documented increased microsatellite levels in urine from a patient who developed an invasive adenocarcinoma within an augmented bladder after cystoplasty [31]. Loss of heterozygosity was identified in both urine and tumor samples from this patient using PCR-based microsatellite analysis, suggesting that urinary microsatellite analysis may be useful as a monitoring tool to detect neoplastic change in patients after augmentation cystoplasty [31].

It is well established that malignant transformation in colonic carcinoma involves the transition from metaplasia, through benign adenoma (without high-grade dysplasia), adenoma with high-grade dysplasia, carcinoma in situ, and finally invasive adenocarcinoma. Tubular adenoma (with or without high-grade dysplasia) is a precursor of gastrointestinal adenocarcinoma, and requires complete surgical excision when encountered in small bowel segments used for urinary tract reconstruction [1]. Tubular adenoma with high-grade dysplasia in an ileal segment used for lower urinary tract reconstruction has not been previously reported to our knowledge. There have been only two previous reports of tubulovillous adenomas in ileal segment after ileocystoplasty, both without high-grade dysplasia [11,12]. There has been only 1 previous report of a tubulovillous adenoma arising in the cecal segment after cecocystoplasty, without high-grade dysplasia [10]. The case herein reported of tubular adenoma with high-grade dysplasia supports the hypothesis that an ileal neobladder may undergo all the morphologic and molecular changes observed in the development of gastrointestinal adenocarcinoma. The previous reports of adenocarcinomas [7-9] and adenomas [11,12], and our finding of a tubular adenoma with high-grade dysplasia in the ileal neobladder support the metaplasia-adenoma-carcinoma sequence as the pathway for carcinogenesis following ileocystoplasty. Therefore, we recommend that patients with transposed intestinal neobladders need to be screened for the development of adenomatous polyps and subsequent carcinomas.

## Conclusion

Patients undergoing augmentation ileocystoplasty are at increased risk of glandular malignancy in the ileal segment. Therefore, clinicians should be aware of the potential of malignant transformation developing in the neobladder when cystoplasty with any kind of intestinal segments are performed. Hence, emphasizing the importance of careful lifelong follow-up, including cystoscopy and urine cytology, of all patients who undergo urinary tract reconstruction with small bowel segments.

## Competing interests

The author(s) declare that they have no competing interests.

## Authors' contributions

HBA participated in the histopathological evaluation, performed the literature review, acquired photomicrographs and drafted the manuscript. AMK and AVP conceived and designed the study, gave the final histopathological diagnosis and revised the manuscript for important intellectual content. All the authors read and approved the final manuscript.
